# Erratum to: Clustering of non-communicable diseases risk factors in Bangladeshi adults: an analysis of STEPS survey 2013

**DOI:** 10.1186/s12889-015-2102-x

**Published:** 2015-09-14

**Authors:** M. Mostafa Zaman, Mahfuzur Rahman Bhuiyan, Nazmul Karim, Moniruz Zaman, Mukhlesur Rahman, Abdul Waheed Akanda, Thushara Fernando

**Affiliations:** Division of NCD, World Health Organization, Dhaka, Bangladesh; Division of Training, Bureau of Health Education, Dhaka, Bangladesh; Present address: Division of Planning and Management, World Health Organization Regional Office for South East Asia, New Delhi, India

## Main text

Unfortunately, the original version of this article [1] contained an error. The figure titles for Figs. 1 and 2 have been interchanged. These figure titles have now been corrected and included correctly in the original article.
